# Genetic Algorithm for Automated Parameterization of Network Hamiltonian Models of Amyloid Fibril Formation

**DOI:** 10.1021/acs.jpcb.3c07322

**Published:** 2024-02-15

**Authors:** Gianmarc Grazioli, Andy Tao, Inika Bhatia, Patrick Regan

**Affiliations:** Department of Chemistry, San José State University, San Jose, California 95192, United States

## Abstract

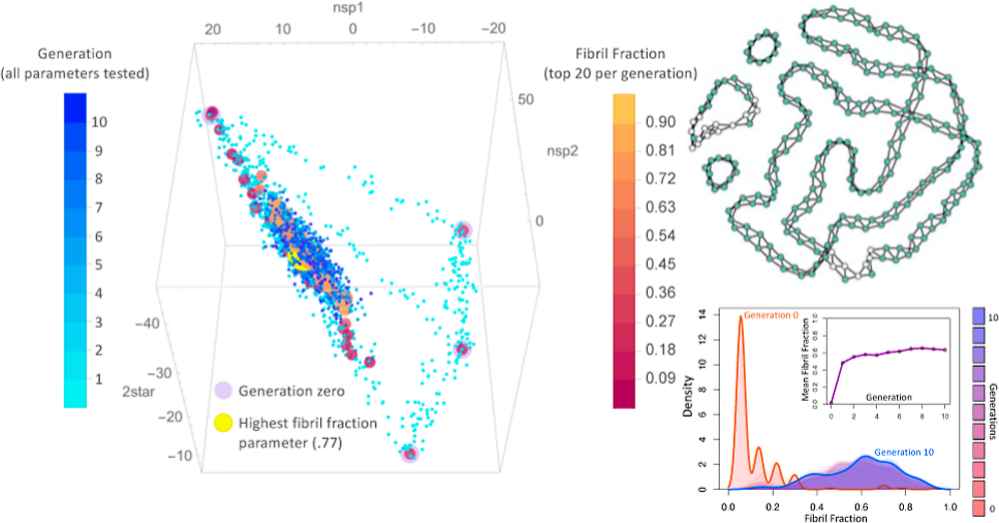

The time scales of long-time atomistic molecular dynamics simulations are typically reported in microseconds, while the time scales for experiments studying the kinetics of amyloid fibril formation are typically reported in minutes or hours. This time scale deficit of roughly 9 orders of magnitude presents a major challenge in the design of computer simulation methods for studying protein aggregation events. Coarse-grained molecular simulations offer a computationally tractable path forward for exploring the molecular mechanism driving the formation of these structures, which are implicated in diseases such as Alzheimer’s, Parkinson’s, and type-II diabetes. Network Hamiltonian models of aggregation are centered around a Hamiltonian function that returns the total energy of a system of aggregating proteins, given the graph structure of the system as an input. In the graph, or network, representation of the system, each protein molecule is represented as a node, and noncovalent bonds between proteins are represented as edges. The parameter, i.e., a set of coefficients that determine the degree to which each topological degree of freedom is favored or disfavored, must be determined for each network Hamiltonian model, and is a well-known technical challenge. The methodology is first demonstrated by beginning with an initial set of randomly parametrized models of low fibril fraction (<5% fibrillar), and evolving to subsequent generations of models, ultimately leading to high fibril fraction models (>70% fibrillar). The methodology is also demonstrated by applying it to optimizing previously published network Hamiltonian models for the 5 key amyloid fibril topologies that have been reported in the Protein Data Bank (PDB). The models generated by the AI produced fibril fractions that surpass previously published fibril fractions in 3 of 5 cases, including the most naturally abundant amyloid fibril topology, the *1,2 2-ribbon*, which features a steric zipper. The authors also aim to encourage more widespread use of the network Hamiltonian methodology for fitting a wide variety of self-assembling systems by releasing a free open-source implementation of the genetic algorithm introduced here.

## Introduction

### Amyloid Fibril Formation and Its Role in Disease

Amyloid fibrils are insoluble fibrous aggregates of proteins that form under a variety of physiological conditions and fall into three categories: pathological amyloids, artificial amyloids, and functional amyloids.^1^ While physiological functions can vary, amyloids share a characteristic cross-β structure.^2^ In contrast to globular proteins, amyloid proteins can flatten, allowing β-sheet-like hydrogen bonding between protein molecules, which leads to protein molecule stacking, resulting in fibril lengthening along a particular fibril growth axis.^3^ Pathological amyloids are associated with a group of degenerative amyloid diseases, including Parkinson’s disease, Alzheimer’s disease, and type-II diabetes.^2,4^ For example, insoluble aggregates of α-synuclein in the form of Lewy bodies inhibits glucocerebrosidase functioning in patients with Parkinson’s disease.^5^ Amyloid fibrils composed of the amyloid-β peptide (Aβ) are strongly associated with Alzheimer’s disease, as Aβ peptides accumulate in the medial temporal lobe of the brain and can have neurotoxic effects, leading to neurodegeneration.^6^ While the importance of amyloid fibrils to biomedical research is well established,^2,4^ and numerous equilibrated amyloid fibril structures have been resolved experimentally,^3,7,8^ elucidating the mechanism of amyloid fibril formation is an ongoing area of research with many open questions.^9−11^ Amyloid fibril growth is characterized by normally soluble proteins undergoing nucleated growth to eventually form insoluble and degradation-resistant aggregates. More precisely, after an initial lag phase, amyloid fibril growth is first initiated from primary nucleation sites, proliferation is then accelerated by secondary nucleation events.^12−14^ Fibril breakage is also believed to further contribute to the total fibril content in a system of aggregating proteins.^12−14^ The known association between amyloids and neurodegenerative diseases has motivated substantial research efforts toward potential treatments. Although existing drugs are used to treat the symptoms of Alzheimer’s disease, for example by either inhibiting the cholinesterase enzyme or antagonists for *N*-methyl d-aspartate (NMDA), a treatment for preventing, reversing, or halting the progression of the disease state is yet to be discovered.^6^ Many disease-modifying treatments are currently undergoing clinical trials to test both prevention and reduction of Alzheimer’s disease. Most of these treatments target Aβ peptides by use of monoclonal antibodies (Aducanumab and Gantenerumab), active immunotherapy (ABvac40), or anti-inflammatories (ALZT-OP1, Azeliragon).^6^ Importantly, there are gaps in knowledge surrounding the mechanism of amyloid fibril formation that are impeding the development of treatments for reversing amyloid diseases. Such gaps include how oligomers, the possible precursors and/or biproducts to amyloid fibril formation, may be formed by many different parallel pathways due to slightly different experimental or physiological conditions.^2,10,11,15−17^

### Coarse-Grained Models for Simulating Protein Aggregation

Although experimental techniques like NMR and X-ray crystallography are commonly combined with atomistic molecular simulations for studying structural dynamics of amyloid fibril structures,^18,19^ such methods are better suited to studying fully equilibrated structures than the transient intermediate structures underlying the mechanism of formation for mature amyloid fibrils. While atomistic classical molecular dynamics simulations can be highly effective for studying molecular motions of individual proteins and nucleic acids,^20−23^ protein aggregation dynamics leading to amyloid fibril formation involves many individual protein molecules interacting on time scales roughly 9 orders of magnitude beyond the reach of typical molecular dynamics simulations (e.g., 1 μs compared to 1 h). Although enhanced sampling techniques for atomistic molecular dynamics simulations can be used to extend the temporal reach of atomistic molecular dynamics simulations,^24−27^ time scale deficits of this magnitude typically require the construction of coarse-grained (CG) models, where degrees of freedom deemed unimportant for characterizing the molecular motions of interest are unified into less detailed fundamental components of the system.^28−32^ Coarse-grained computational models of protein aggregation are useful for suggesting potential mechanisms for amyloid fibril formation at a higher level of detail than what is directly accessible to experiments while remaining grounded in experiments due to computed observables being consistent with experimentally measured observables.^11,13,33−38^ Most coarse-grained models of molecular self-assembly employ a bottom-up approach, wherein the underlying physics driving monomer dynamics at an atomistic level of detail is coarse-grained down to a set of mechanical degrees of freedom deemed essential by the builder of that model. Such approaches yield fibril structures as emergent phenomena from the modeled monomer dynamics. One well-known coarse-grained model of aggregation of the bottom-up variety is that of Šarić et al.,^39^ in which entire proteins are represented as oblong particles, and configurational changes are modeled as patches of attraction that are shifted from the side of the particle to the tip of the particle. This model has been used to propose a mechanism of amyloid fibril formation whereby amyloid fibrils are preceded by the formation of prefibrillar oligomers.^39^ Another coarse-grained modeling approach of a similar level of coarseness to the aforementioned work by Šarić et al.,^39^ i.e., whereby the fundamental interacting bodies are entire protein molecules, is the network Hamiltonian model (NHM),^13,29,36,40,41^ which is the focus of the present work.

Conversely to bottom-up approaches, NHMs operate via a top-down strategy, where exponential random graph models (ERGMs) are used to directly simulate fibril topology formation.^29^ This approach enables the extraction of a statistical mechanical description of the intermolecular interactions driving the emergence of the known aggregate topology, once the model parameters have been tuned to reproduce network structures that match those measured using experimental techniques like NMR, X-ray crystallography, etc.^29^ The NHM methodology offers a complementary perspective to bottom-up techniques by not only reducing computational cost but also leveraging the congruence between the construction of normalizing constants in ERGMs and partition functions in statistical mechanics to enable direct derivation of a statistical mechanical description of the system of aggregating molecules during parametrization.^29^ Another feature of NHMs is that their parsimonious description of the few body intermolecular effects driving the formation of higher order structure facilitates direct comparison between network Hamiltonians representing different aggregating systems (example given in the final paragraph of the Results and Discussion section). One particularly interesting mechanistic insight into amyloid fibril formation, which was brought to light using network Hamiltonian models by Yu et al.,^41^ is that fibrillar fraction growth curves for the simple 1-ribbon fibril topology exhibit steady gentle growth compared to the higher ordered structures that display a sharp increase in fibril formation after the initial lag phase. Such developments offer interesting targets for experimental probes involving comparisons between computed fibril fraction growth curves and fibril growth kinetics data obtained via dye-binding fluorescence assays.^42−44^ Though not an exhaustive list, some of the leading coarse-grained modeling approaches that have been applied toward studying amyloid fibril formation include: single bead per amino acid models,^11,45,46^ midresolution models (multiple beads per residue),^10,15,47−50^ the UNRES model,^37,51^ the Martini model,^52,53^ lattice models,^34,54,55^ and network Hamiltonian models.^13,29,36,40,41^

### Network Hamiltonian Simulations of Amyloid Fibril Formation

Network Hamiltonian models are highly computationally efficient coarse-grained (CG) molecular simulations, which are capable of reproducing multiple known experimental observables (e.g., topological structures measured via NMR and fibril formation growth curves from dye-binding fluorescence kinetics assays^13,28^), and can provide insights into the complex interactions between proteins that lead to the formation of amyloid fibrils. By sacrificing atomic-level details, these models can capture the essential large-scale interactions and dynamics that drive amyloid aggregation. This facilitates the discovery of assembly mechanisms and provides insights into the structure and stability of amyloid fibrils. These simulations capture essential interactions and dynamics for noncovalent bond formation and breakage between aggregating proteins by directly modeling the graph structure dynamics. It is important to note that network Hamiltonian models (NHMs) of this form are exclusively topological models, in that they offer massive gains in computational speedup by directly simulating changes in noncovalent bonding between interacting protein molecules without accruing computational costs associated with updating explicit molecular positions. Network Hamiltonian simulations can offer valuable insights into the thermodynamics, kinetics, and structural transitions involved in amyloid fibril assembly, which can aid in identifying targets for the design of potential therapeutic strategies. There are substantial technical challenges inherent to parameter selection for NHMs, as the parameter space is fraught with discontinuities and nonlinearities.^28^ Although prior NHMs have been successfully parametrized to produce a variety of aggregate structures,^13,28,36,40,41^ their parametrization was carried out using a combination of different optimization methods and manual search,^28^ as well as standard practices used for social networks, as demonstrated by Hunter et al.^56^ In contrast, here we present a single fully automated methodology for parametrizing an NHM to self-assemble into aggregate states displaying a maximal periodic structure, for any user-defined periodic structures (e.g., the 5 amyloid fibril structures demonstrated here).

## Methods

### Network Hamiltonian Theory

**Figure 1:**
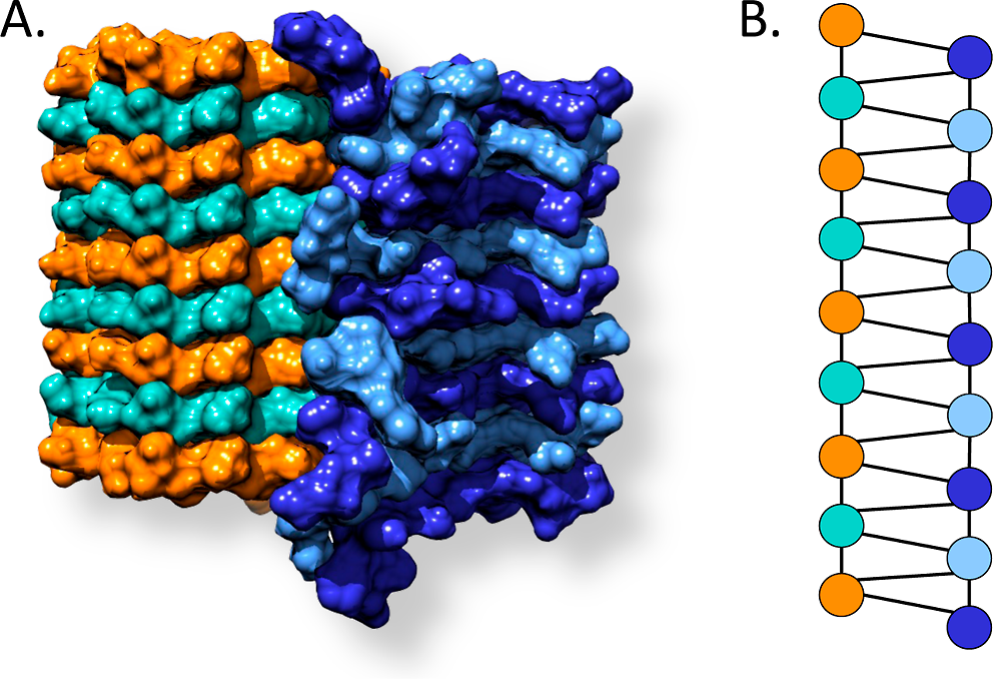
(A) Rendering of the NMR structure of amyloid-β (Aβ42).^7,59^ (B) Graph representation of that same structure with the nodes colored and positioned to highlight the mapping of each protein chain to a particular node,^29^ though the nodes are only distinguishable by the graph structure in the model.

Network Hamiltonian models are a type of coarse-grained molecular simulation that can be used to model amyloid fibril self-assembly, or other types of aggregation events,^13,29,40,41,57^ and are built within the framework of exponential-family random graph models (ERGMs).^58^ The principal objects in network Hamiltonian simulations are graphs (networks), where each node represents a molecule in the aggregating system and a pair of nodes share an edge if a pair of molecules in the system share a noncovalent bond. An example of mapping an experimentally determined amyloid fibril structure is shown in Figure 1, where Grazioli et al.^29^ used a free energy scoring methodology to quantitatively map the structure of an amyloid-β fibril resolved using NMR spectroscopy (PDB ID: 5KK3(7)) to a graph representation.

Central to the network Hamiltonian formalism is the expression for the probability of observing a particular graph *g* from the set of all possible graphs  given a particular set of sufficient statistics *t*, a vector of parameters ϕ, and temperature *T* shown in eq 1
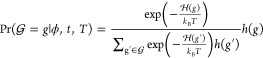
1where  is the network Hamiltonian, *k*_B_ is Boltzmann’s constant, and *h*(*g*) is the reference measure.^29^ The expression is isomorphic with the Boltzmann distribution, the key difference being that instead of the Hamiltonian being a function of the positions and momenta of the particles in the system it is a function of the graph structure, i.e., which particles share a noncovalent bond and which do not. The network Hamiltonian itself is the sum of a set of sufficient statistics *t* each multiplied by its respective real number parameter ϕ, as shown in the expanded form in eq 2
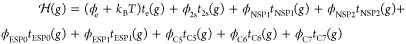
2

**Figure 2:**
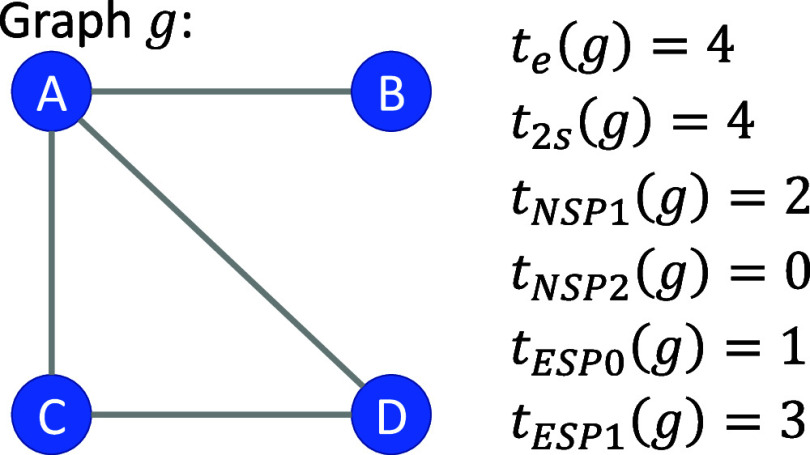
Six of the sufficient statistics utilized in the present work are demonstrated on a simple example of a graph *g*. Each function ϕ_*X*_(*g*) returns the number of times subgraphs of type *X* occur in the graph *g*.

Each term of the network Hamiltonian includes a real valued coefficient multiplied by a sufficient statistic, *t*_*X*_(*g*), which is a function that returns the number of subgraphs of type *X* that occur in the graph *g*. As an example, consider the graph in Figure 2. The simplest statistic, the edges statistic *t*_e_(*g*), returns the number of edges in the graph. The 2-star statistic *t*_2s_ returns the number of times a central node shares edges with two other nodes. Intuition for the other sufficient statistics can be gleaned by considering their applications to the simple graph shown in Figure 2. For the graph in Figure 2, the single null shared partner statistic *t*_NSP1_(*g*) = 2 because nodes *B* and *C* have a shared partner *A* but do not share an edge with each other, as is the case with nodes *B* and *D* with shared partner *A* (i.e., 2 instances of a null between nodes with one shared partner). The edgewise shared partner zero statistic, *t*_ESP0_(*g*), is equal to 1 in Figure 2 because there is only one instance where two nodes sharing an edge do not share any partners (nodes *A* and *B*). Although the cycle statistics [*t*_C5_(*g*), *t*_C6_(*g*), and *t*_C7_(*g*)] would all return zero in the example in Figure 2, a 3-cycle statistic, *t*_C3_(*g*), would return 1, as one 3-membered ring is present. Physical intuition for the parameters of the network Hamiltonian, i.e., the coefficients ϕ_X_ in each term of the network Hamiltonian, can be gained by interpreting a negative parameter value as indicating that the formation of the subgraph corresponding to it is respective sufficient statistic [*t*_X_(*g*)] has an exothermic effect on the system, while positive values indicate an energetic cost or endothermic effect on their formation, and zero valued coefficients indicate that the system is indifferent to the formation of that subgraph.

The compact expression for a single parameter vector ϕ containing the set of network Hamiltonian parameters, for which the genetic algorithm featured in the present work is designed to optimize, is shown in eq 3
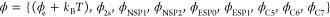
3

It should also be noted that the physically motivated ϕ representation of the network Hamiltonian (systems equilibrate toward lower energy states) must be translated into the more statistically motivated θ forms (systems equilibrate toward higher likelihood states) prior to carrying out simulations using the ERGM software package (where θ = −ϕ/(*k*_B_*T*))^58^
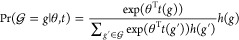
4where θ^T^*t*(*g*) is simply the set of sufficient statistics multiplied by the transpose of the parameter vector. For additional details on the network Hamiltonian methodology, please see both the main text and the Supporting Information from Grazioli et al. 2019.^29^

There are numerous sufficient statistics that can be incorporated into a NHM,^58^ the choice of which can be influenced by anything from user insights into known microscopic interactions between interacting protein molecules to empirical approaches whereby sufficient statistics found to produce a known higher order structure as an emergent property are used to develop mechanistic details based in few body interactions. The set of sufficient statistics employed in the network Hamiltonian models for the 5 fibril topologies demonstrated here were chosen based on previously established models,^13,29,41^ and although the present work is focused on introducing our genetic algorithm and demonstrating its efficacy by (re)tuning the coefficients for network Hamiltonians employing this same choice of sufficient statistics, it is worth briefly addressing strategies and physical justifications for choosing a particular set of sufficient statistics to be included in a network Hamiltonian model. As per previous work,^13,29,41^ the terms can be described in terms of energy as follows. *t*_e_(*g*), the number of edges in *g*, establishes the baseline first order energetic cost of an edge in isolation. *t*_2s_(*g*), the number of occurrences of a monomer bound to two other monomers, can be thought of as the energetic cost of forming a new bond to a given monomer that is brought on by all existing bonds to that monomer. For example, a *t*_2s_(*g*) term with a positive coefficient in the network Hamiltonian could be interpreted as an allosteric effect whereby the free energy of binding to a protein is diminished with each subsequent binding event. *t*_NSP1_(*g*) and *t*_NSP2_(*g*), the null shared-partner terms, and the cycle terms (*t*_c5_(*g*) – *t*_c7_(*g*)), are multibody interactions that can be thought of as higher-order rigidity effects (e.g., an energetic penalty for forming 5-cycles can be interpreted as structural resistance inhibiting closure of a 5 membered ring). Finally, the edgewise shared-partner terms, *t*_ESP0_(*g*) and *t*_ESP1_(*g*),^60^ are related to triadic closure, i.e., the tendency of monomers that are both bound to a common partner to bind to each another.

### Description of the Genetic Algorithm Used to Parametrize the Models

**Figure 3:**
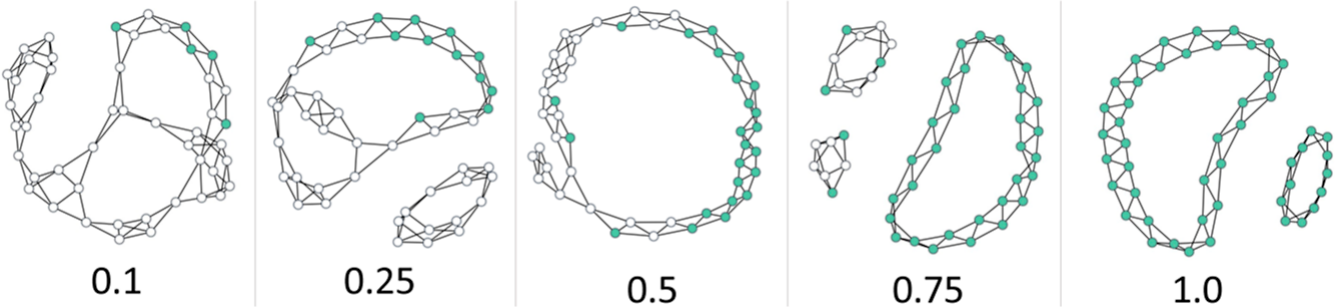
Five examples of graphs produced by 48 node simulations of models parametrized to produce *1,2 2-ribbon* type fibril structures, with the fibril fraction for each below the graphs.

The use of AI and machine learning (ML) to optimize, interpret, and guide molecular simulations has become ubiquitous in fields ranging from biophysics, materials science, drug discovery, and others.^27,28,57,61−69^ Genetic algorithms are a class of unsupervised machine learning algorithms that are inspired by the process of natural selection and genetics in biological systems.^70^ They proceed via an iterative process involving a population of candidate solutions that undergo recombination and mutation to create new offspring solutions. A selection process based on the fitness of each solution, or model, is used to determine which solutions survive into the next generation. Just as evolutionary pressures are a powerful driver for shaping the evolution of organisms, the metric used to determine each candidate model’s ability to survive selection and go on to participate in breeding the next generation of models is of central importance to shaping the evolution of candidate models in a genetic algorithm. Because the goal here is to discover network Hamiltonian models that can maximally and reliably simulate the self-assembly of amyloid fibril network structures, we define a metric for amyloid fibril production called fibril fraction,^29^ which is defined simply as the number of nodes in the system that make up part of a region of perfect amyloid fibril divided by the total number of nodes in the simulation (e.g., in Figure 3). Calculating fibril fraction is carried out using custom scripts in the R statistical computing environment, along with a variety of R packages,^71−75^ using approaches standard to the network Hamiltonian methodology.^13,28,29,40,41,57^ The R script used to calculate fibril fraction for the present work (*fibril_assay.R*^76,77^) was constructed with the goal of rewarding parameters that produce longer fibrils; thus, fibrillar nodes that belong to the interior of an amyloid fibril structure were prioritized in fibril fraction calculations for the present work. Although this stringent metric can lead to a slight undercounting of fibrillar nodes (e.g., the 0.25 fibril fraction in Figure 3), the strategy was deemed fit-for-purpose given the success of the genetic algorithm in optimizing for models of high fibril fraction using this metric (demonstrated in the Results and Discussion section).

**Figure 4:**
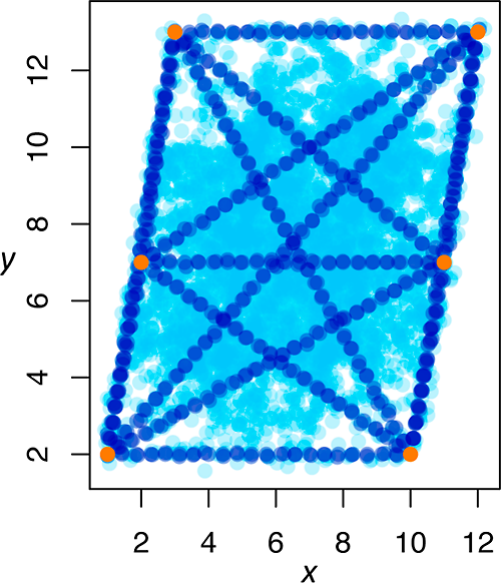
Demonstration of the breeding function on a 2 parameter model (*x* and *y*) showing 3 generations: 1 (orange), 2 (dark blue), and 3 (light blue). Note that the region of parameter space that lies within the convex hull of generation 1 is thoroughly explored by the third generation. Both the density of points and the variance in the noise can be altered with different hyperparameter choices.

Also important in the creation of a genetic algorithm is the definition of how the exchange of genes, i.e. breeding, is carried out. In the present case, intuition gained from studying the stability of graph structures under a particular parametrization of an ERGM was leveraged.^41^ Specifically, it was observed that higher fibril fraction-producing parameters are often found within the convex hull of less successful parameters.^41^ Building on this observation, a breeding process was created for the present work whereby child parameters are created by first connecting all possible breeding pairs with N-dimensional lines in a parameter space (one dimension for each sufficient statistic in the model), then placing child parameters on an evenly spaced grid along each line (referred to as linear children), then making copies of the linear children with noise added (referred to as mutant children). A visual demonstration of the breeding process is shown in Figure 4.

**Figure 5:**
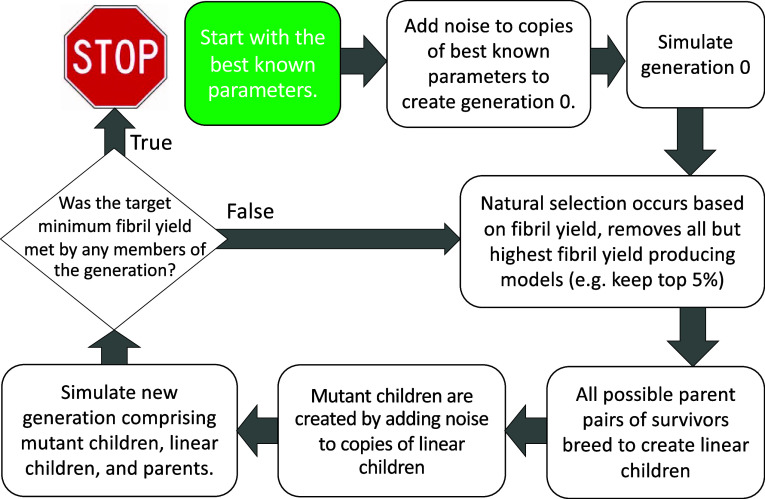
Schematic showing an overview of the genetic algorithm used to autonomously search the parameter space for a given set of sufficient statistics for models that will maximize the fibril fraction for a given topology. Users can create best known parameters through a variety of methods, e.g., randomly sampling parameters in a region where models capable of producing aggregate structures similar to the target have been produced and then running simulations with those parameters to determine the most successful randomly chosen parameters. The breeding process can be thought of as placing points in parameter space along *N* dimensional lines connecting previously successful parent points in the parameter space (where *N* is the number of sufficient statistics in the model), and is described in greater detail in this section. Additionally, the hyperparameters used to tune the algorithm are summarized in Table 1.

A schematic illustrating how our genetic algorithm for optimizing network Hamiltonian models for maximal amyloid fibril production operates is shown in Figure 5, but there are some additional subtleties that bear mentioning. For example, although genetic algorithms are inspired by natural selection, there are circumstances where it is advantageous for genetic algorithms to depart from some of the constraints typically found in biological natural selection. For example, in the event that a new generation does not produce a single simulation that outperforms the best fibril fraction from that generation’s parent generation, the entire new generation is removed from the gene pool and the parent generation repeats the breeding process in the hopes that a new generation of children will outperform them. Including a copy of all breeding parents in each new generation is also done because, in the event that a consistently top performing parameter arises, it can become immortal. Another departure from typical biological natural selection is that, in order to ensure more complete sampling of parameter space, all possible pairs of survivor points in the parameter space are given the opportunity to breed. Because this condition leads to proliferation on the order of *N*^2^, where *N* is the number of breeding parents (potentially challenging for available computing resources), a hyperparameter was created for the algorithm called *childMax*, whereby, if a breeding pair produces more children than the value of *childMax*, that number of child parameters is selected at random from the full set of children to go on to the simulation step where fibril fraction is assessed. Further discussion of hyperparameters, such as *childMax*, is continued in the following section and summarized in Table 1.

**Table 1:** Table Highlighting Some of the Features Included in the Genetic Algorithm Code

feature	purpose	typical parameter values
reps	number of simulations used to calculate mean fibril yield	16
noiseVarInit	initial variance of noise used to generate mutants	1.0
topFraction	top fraction of models in a generation that survive selection	0.25
maxSurvivors	sets hard limit to the number of survivors; if exceeded, will randomly select from top fraction survivors	20
fixEdge/fixEdgeValue	can be used to fix edge value during evolution	100
smartVar	if a new generation does not outperform the previous, smart variance increases the variance to broaden the search	true
smartPts	if a new generation does not outperform the previous, smart points increases the number of mutant offspring for each breeding pair by one	true
useLineDensity/minLineDensity	ensures that a minimum density of offspring points are created between each breeding pair	true/1.2
childMax	sets a hard limit to number of children produced by each breeding pair; if exceeded, survivors are chosen randomly	100

### Choosing the Hyperparameters for the Genetic Algorithm

Most machine learning algorithms require users to choose a set of hyperparameters, i.e. training parameters that are chosen prior to beginning the learning process,^78^ and the genetic algorithm introduced here is no different in that regard (Table 1 gives an overview of key hyperparameters). We begin the discussion with the *reps* hyperparameter, which determines the number of simulations run for each candidate model when calculating the mean fibril fraction (the measure used to govern the natural selection process). Notably, it was observed that running the genetic algorithm with lower *reps* values can lead toward favoring regions of parameter space that produce multimodal distributions that include a high-yield mode, as fewer repetitions make the mean fibril fraction more susceptible to getting skewed by a small number of uncharacteristically high values. Running larger *reps* values will ensure that the parameters chosen to survive natural selection are more consistent performers, but, of course, this is at the cost of longer compute times. The hyperparameter *noiseVarInit* determines the initial variance in the noise used to add noise to linear children when creating the mutant children. Choosing larger *noiseVarInit* values allow for broader searches, but this comes at the risk of allowing the search to wander off into unproductive regions. While the *noiseVarInit* hyperparameter determines the initial variance, *smartVar*, i.e. smart variance, instructs the algorithm to broaden the search by increasing the variance when the latest generation of children does not outperform their parents. Likewise, the hyperparameter *smartPts*, i.e. smart points, instructs the algorithm to generate more children each time the latest new generation does not outperform the parents. The hyperparameters *useLineDensity* and *minLineDensity* were created in order to ensure more uniform sampling by allowing pairs of parent points separated by further Euclidian distances in parameter space to produce more children than those separated by shorter distances. An additional feature that is coded into the algorithm is that parent pairs of points in parameter space that lie so close together that the chosen *minLineDensity* value would imply less than one child between them are deemed too closely related to breed. This design choice, which essentially avoids inbreeding, was made in order to prevent wasting computing resources running simulations on child models that are highly similar to their parents. The rest of the hyperparameters can be easily understood via Table 1.

## Results and Discussion

The focus of this section is to demonstrate the capabilities of the genetic algorithm introduced in the present work by using it to find parameters that maximize amyloid fibril production in network Hamiltonian simulations for each of the five experimentally observed amyloid fibril topologies.^29^ We begin by demonstrating that the genetic algorithm is able to find high fibril fraction-producing parameters (up to 0.77) for the 2-ribbon topology, given an initial generation of four models with single draw fibril fractions of just 0.04, 0.02, 0.02, and 0.02. Next, we demonstrate the capabilities of the genetic algorithm by beginning the evolution from a zeroth generation centered at previously published model parameters. The genetic algorithm was able to find substantially higher fibril fraction-producing models for 3 of the 5 fibrillar topologies observed in nature, including the *1,2 2-ribbon*, which is the most naturally abundant amyloid fibril topology^29^ (often referred to as a steric zipper structure^79,80^).

### Genetic Algorithm Performance Initialized from Randomly Chosen Model Parameters

**Figure 6:**
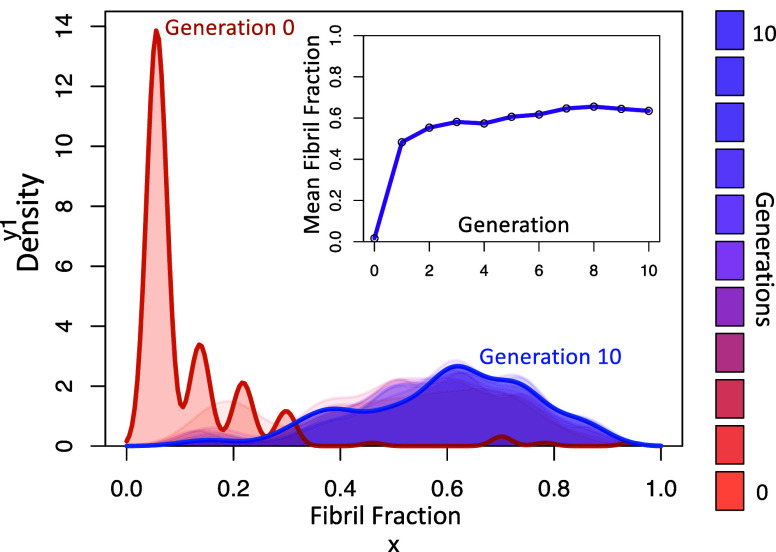
Fibril fraction distributions produced by 200 repetitions of the best 2-ribbon model from each generation beginning with the best randomly parametrized model (generation 0). Inset shows the mean fibril fraction produced by each generation. The parameters are the best of each generation shown in parameter space in Figure 7.

**Figure 7:**
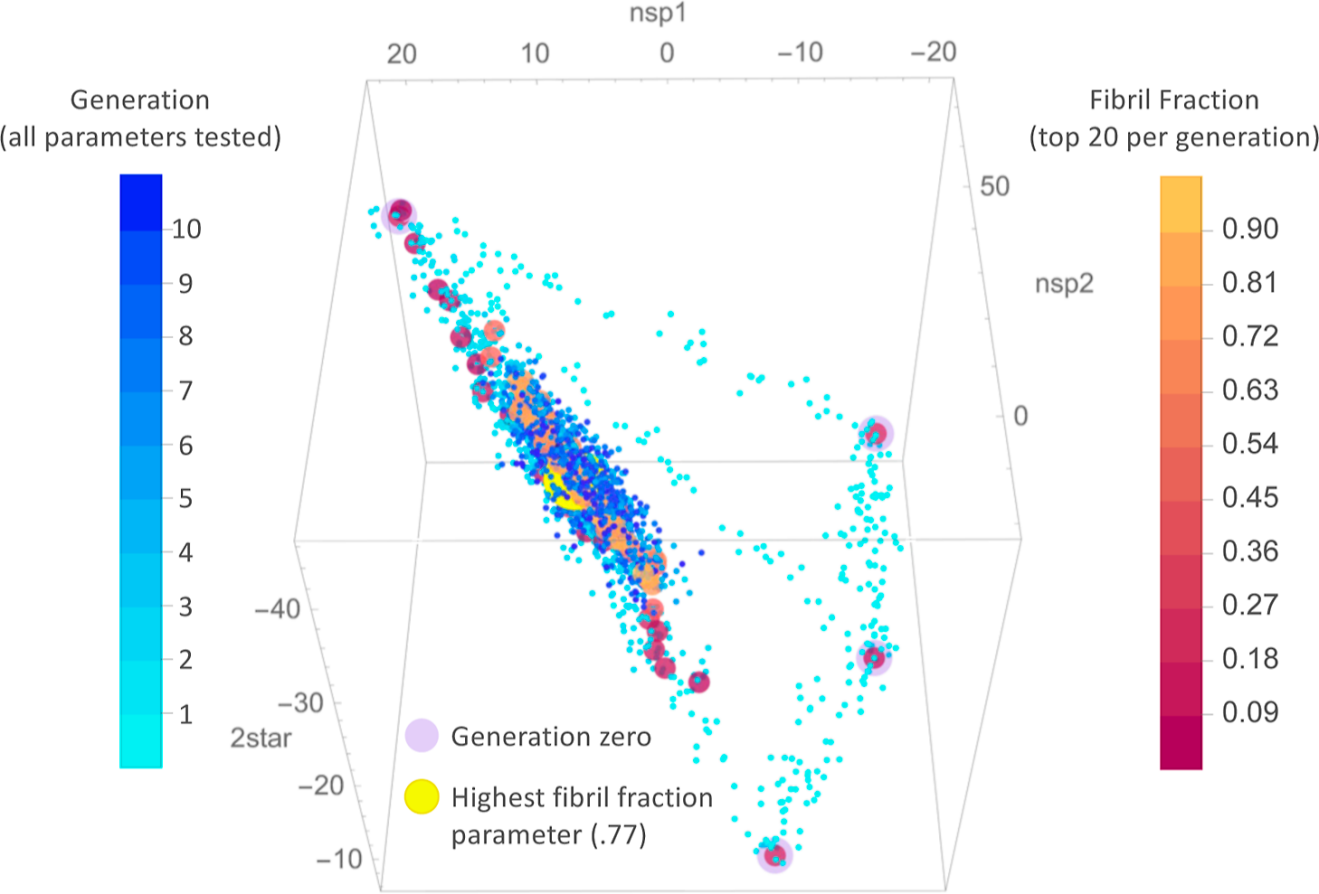
This 3D point plot shows the evolution of different generations of models as the genetic algorithm converges on a region of parameter space that produces maximal fibril yield for 2-ribbon type amyloid fibril structures. The cool color palette signifies the generation, with the deepest blues representing the latest generations. The warm color palette signifies the fibril yield for the top 20 fibril-producing parameters for each generation, where colors closest to yellow indicate the highest fibril yields. Four larger pale pink dots, which are referred to as *generation 0*, are used to highlight the four initial parameters found via a brute force approach. The parameter discovered by the genetic algorithm that produced the highest mean fibril yield (0.77) is shown as a large yellow dot. Fibril yields indicated are the mean of 16 repetitions for all parameter values tested. It should also be noted that, although this model is a 4 parameter model, this particular search was carried out with the edge parameter fixed at 100, reducing the search to 3-dimensions.

Here, we test the conjecture, based in prior works on network stability,^41^ that searching within the convex hull and surrounding regions in the parameter space bounded by low fibril fraction-producing parameters can be fertile ground for identifying higher fibril fraction parametrizations of a given network Hamiltonian model. Given this motivation, the genetic algorithm was used to discover 4-parameter network Hamiltonian models with sufficient statistics *t*_e_(*g*), *t*_2s_(*g*), *t*_NSP1_(*g*), and *t*_NSP2_(*g*) that produce maximal fibril fractions for the *2-ribbon* topology. The process was initiated by first sampling a sphere of 10,000 randomly distributed points in a 3-dimensional parameter space (the edge parameter was held constant at 100 for visualization purposes). Single simulations were run for all parameters, and though most produced zero fibril fraction, a select few did produce some fibrillar structures. In order to showcase the robustness of the genetic algorithm, the four lowest nonzero fibril fraction parameters were selected as generation zero (fibril fractions of 0.04, 0.02, 0.02, and 0.02). After completing 10 generations of evolution using the genetic algorithm, the process converged upon producing multiple generations capable of consistently producing fibril fractions in an excess of 0.6 (Figures 6 and 7). It should also be noted that, in order to reduce the 4-D parameter space to a visualizable 3-D space, the edge parameter was held constant at θ_e_ = 100 throughout the evolution for this demonstration.

### Genetic Algorithm Performance Initialized from Previous Model Parameters

**Figure 8:**
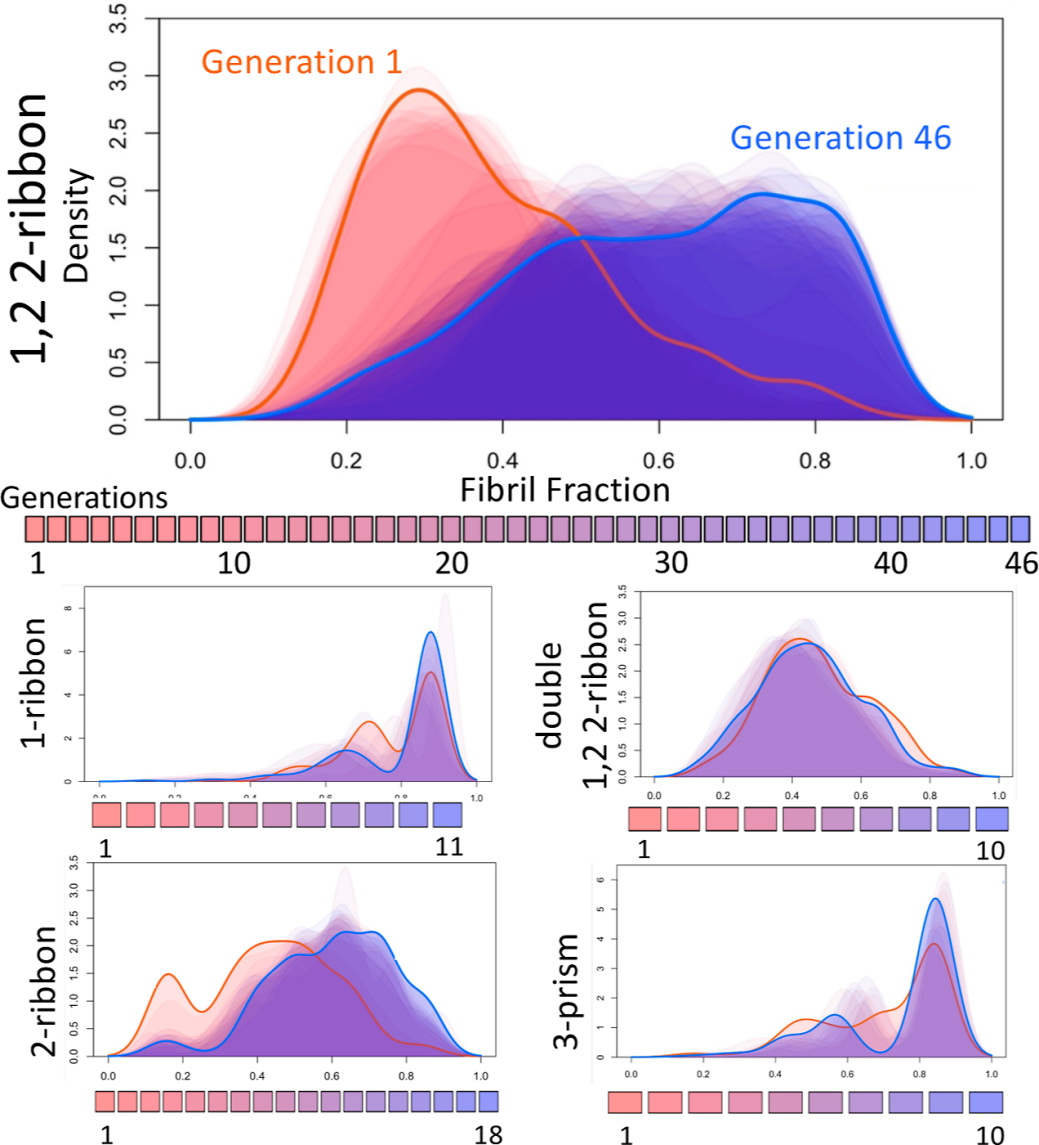
Population density functions for the best parameter produced by each generation of amyloid fibril-producing network Hamiltonian models. Simulations for each parameter were repeated 200 times.

While the previous section demonstrated the power of the genetic algorithm to optimize network Hamiltonian parameters from very weak initial guesses; here, we show that the genetic algorithm can also be used to further optimize previously discovered network Hamiltonian models.^13,29^ In these applications, the zeroth generation was created by centering a randomly distributed point cloud in the parameter space around parameters for each fibril topology, which were previously published by Grazioli et al.^29^ The results of these parameter evolutions, carried out on a 48 node system, are shown in Figure 8. The evolution of the *1,2 2-ribbon* is highlighted, as it is both the amyloid fibril topology most commonly observed in nature,^29^ and it exhibited a substantial increase in fibril fraction as a result of applying our genetic algorithm. The distributions were generated by carrying out 200 simulations on the highest fibril fraction-producing parameter from each generation.

**Figure 9:**
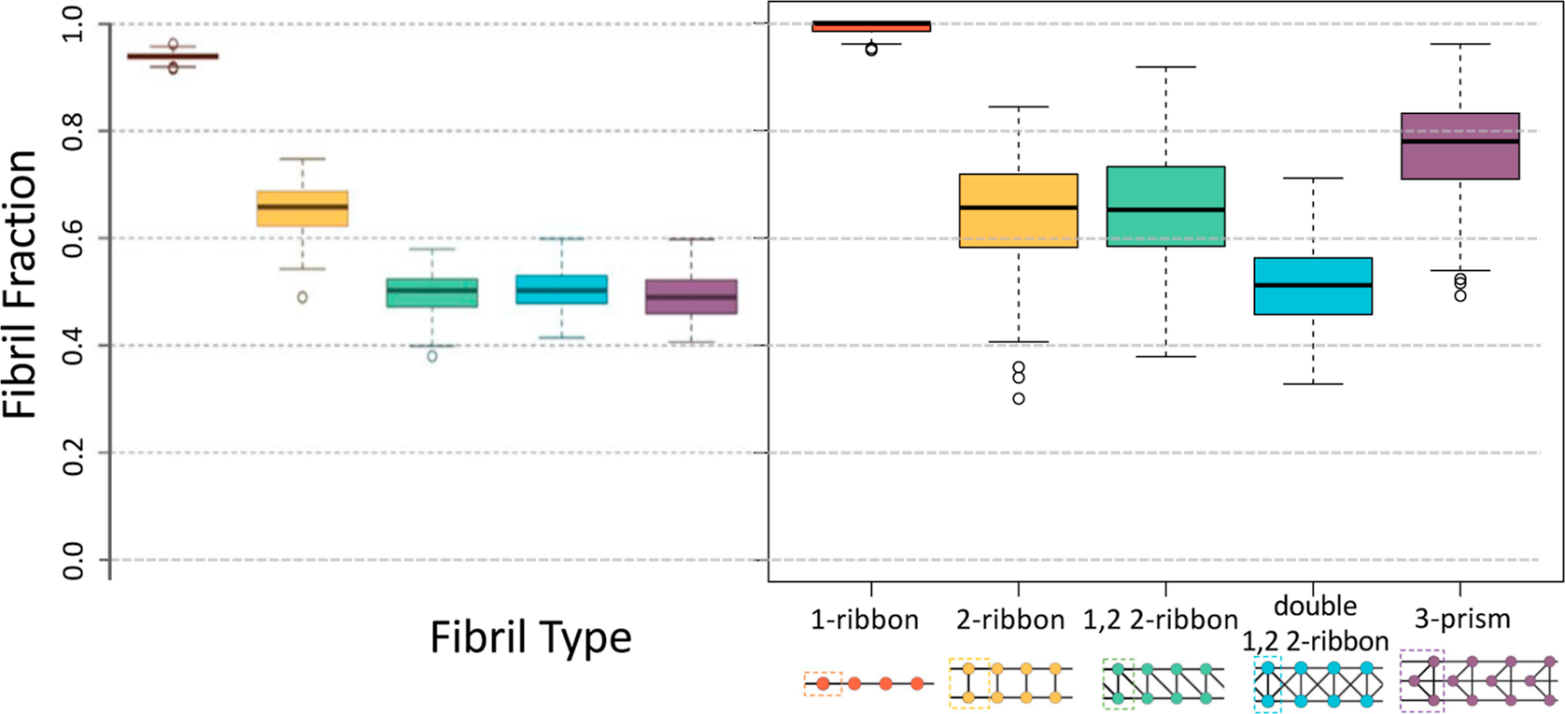
Box plots comparing fibril fractions previously reported by Grazioli et al.^29^ (left) with fibril fractions observed in simulations produced by parameters discovered by the genetic algorithm introduced in the present work (right). The simulations for the present work were carried out on a 256 node system, and repeated 200 times for each of the 5 different models. Maximal fibril fraction draws for each of the 5 models are shown in Figure 10.

**Figure 10:**
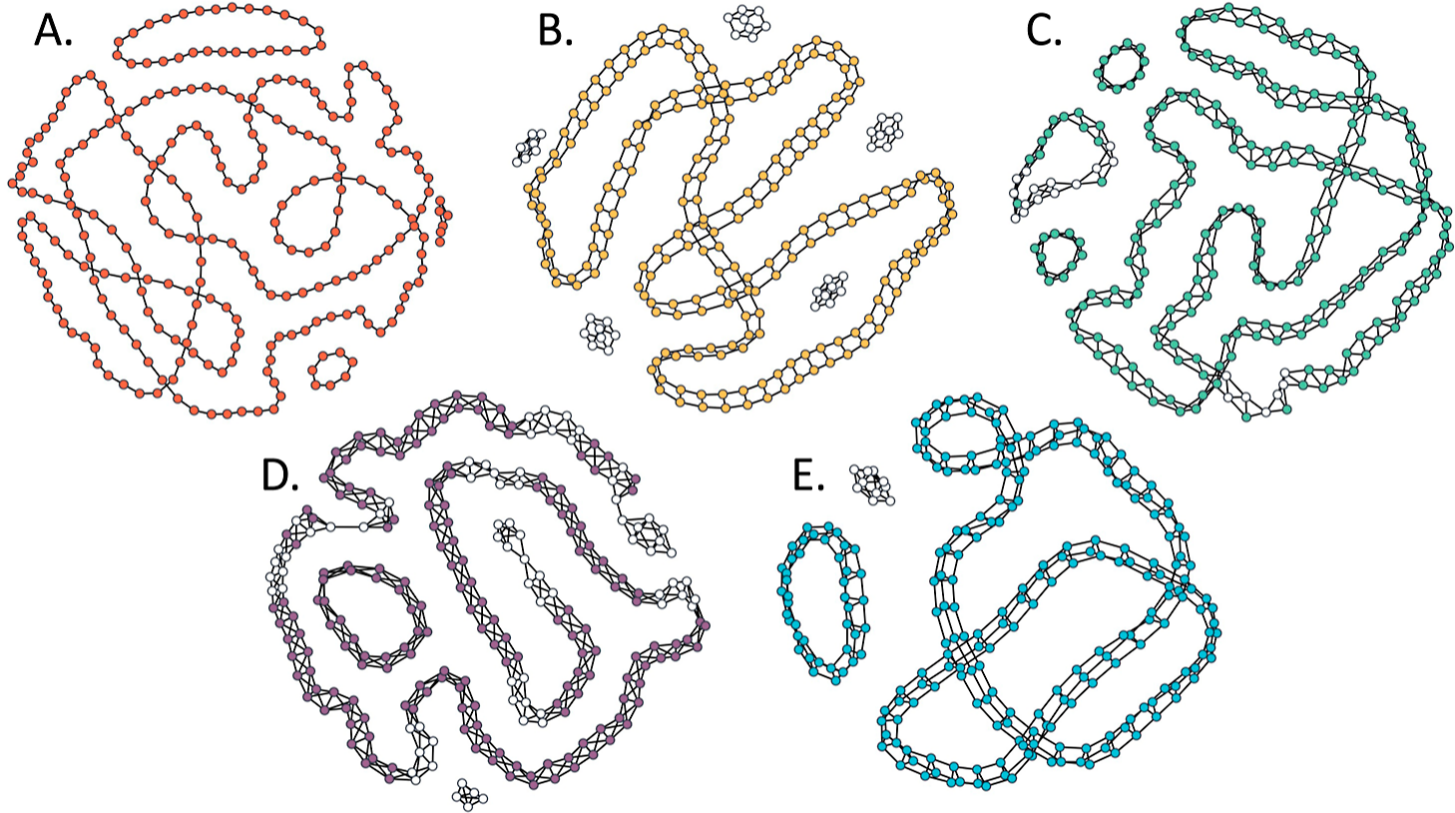
Plots of the graphs produced by simulations yielding the highest fibril fraction for a system of 256 nodes: (A) *1-ribbon* (fibril fraction = 1.0), (B) *2-ribbon* (fibril fraction = 0.8438), (C) *1,2 2-ribbon* (fibril fraction = 0.9180), (D) *double 1,2 2-ribbon* (fibril fraction = 0.7109), and (E) *3-prism* (fibril fraction = 0.9609). Fibrillar nodes are shown in color and nonfibrillar nodes are displayed in white. These graphs display the highest fibril fractions produced for 200 repetitions of each of the 5 models.

In order to ensure that the genetic algorithm did not converge on ideal parameters for smaller systems that might not scale to larger systems, the best generations from the 48 node evolutions for each of the 5 fibril types were then used as the first generation for an additional evolution sequence carried out on a 256 node system. It should be noted, however, that scaling network Hamiltonian models to accommodate different system sizes can be accomplished by simply scaling the edge parameter via a Krivitski offset.^29,81^ The log(*N*) term serves to attenuate the edge term depending on the number of particles in the system, allowing for better scalability in applying the same model to different sized systems. Intuition for this offset can be gained by considering that the increase in baseline likelihood of bond formation between two proteins in a system as a function of the number of proteins in the system should diminish when the system size increases proportionally with volume, as bond formation is limited to pairs of proteins that are spatially accessible to each other within a given time step. In this case, the offset would be ϕ_e_ + log(48) – log(256) because the ϕ_e_ – log(48) is implicit for all edge parameters generated by the genetic algorithm evolving on a 48 node system. The highest fibril fraction-producing parameters for each of the 5 fibril types were then used to run 200 repetitions of each simulation. The results of these simulations are summarized in Figures 9 and 10. Although the previously reported parameters for the *2-ribbon* and *double 1,2 2-ribbon* amyloid fibril topologies seem to have already been well optimized, these results show a marked improvement for the *1-ribbon*, *3-prism*, and *1,2 2-ribbon* topologies. The parameters discovered by the genetic algorithm in the present study that produced the highest fibril fraction are given in Table 2. It is important to note that the parameters are reported in the more physically motivated ϕ form, where θ = −ϕ/(*k*_B_*T*), and, for the sake of simplicity, *k*_B_*T* = 1. Thus, in order to use these parameters to run simulations using the ERGM package for R,^71,74,75^ where the simulation parameters must be in the θ form, set θ_e_ = −(ϕ_e_ + 1 – log(*N*)) (where *N* is the desired number of nodes to be simulated), and θ_*X*_ = −ϕ_*X*_ for all other sufficient statistics *X*.

**Table 2:** Table Displaying the Parameters (ϕ) with the Highest Mean Fibril Fractions Discovered by our Genetic Algorithm

fibril type	edges	2-star	NSP1	NSP2	ESP0	ESP1	5-cycle	6-cycle	7-cycle
1-ribbon	–107.22	37.33	1.35	0	0	0	0	0	0
2-ribbon	–102.28	25.2	–0.44	–5.56	0	0	0	0	0
*1,22-ribbon*	–158.21	27.28	1.87	–7.12	6.59	0	0	0	0
*double1,2 2-ribbon*	–471.95	65.31	–0.25	–23.99	75.18	62.03	0	0	0
3-prism	–193.35	38.13	–5.77	–14.7	–2.51	–11.14	0.12	0.69	–0.02

A key value offered by the NHM methodology is that, by directly casting the model in a purely topological description from the outset, the parametrized NHMs lend themselves to straightforward interpretation of how the different few body interactions drive the self-assembly of the higher order structure by comparing the sign and magnitude of specific parameters between models. For example, in comparing the parameters optimized by the genetic algorithm in Table 2 for the 2-ribbon vs the 1,2 2-ribbon, we note that while the energetic penalty for forming 2-stars is comparable, the simple 2-body energetic advantage for bond formation represented by the edge parameter is more than 50% stronger in the 1,2 2-ribbon model. One potential interpretation of this observation could be that in comparing two sets of aggregating proteins with similar attenuation of bond strength with each additional bond formed (the 2-star effect), a stronger baseline 2-body attraction (the edge effect), whether due to differences in amino acid sequence or solvent conditions like ionic strength, can contribute to the system favoring the more compact *1,2 2-ribbon* structure. Such comparisons between models can be used to develop hypotheses for explaining known amyloid fibril behavior, such as addressing the question of why the same protein monomer can produce different fibril structures given different solvent conditions.^29,82^ Although cyclic fibrillar structures have been observed in both experimentally^83,84^ and in prior simulations of amyloid fibril formation,^29,48^ it is notable that cyclic fibrillar structures are prevalent in the network Hamiltonian models presented herein. This is to be expected, however, as the utility function maximized by the genetic algorithm for these models rewards the models that generate higher order structure consistent with the interior of the amyloid fibril topology. While this design choice in the utility function encourages the evolution of models that grow long fibrillar structures, it also discourages free ends, leading to the ends of the fibrillar networks joining to form a cycle. This is somewhat analogous to how one would not expect to observe surface effects in a computational fluid dynamics model optimized for bulk simulations of fluids using periodic boundary conditions.

## Conclusions

Here, we have demonstrated that our genetic algorithm for automated discovery of network Hamiltonian models (NHMs) can successfully produce models capable of self-assembling into experimentally observed protein aggregate structures. The results of the present study also offer an improved model for the most naturally abundant amyloid fibril topology, the 1,2 2-ribbon, which is reflected in the steric zipper motif that is widely reported in the literature.^79,80^ Further, the network Hamiltonian model (NHM) methodology offers a unique perspective in the study of molecular self-assembly and supramolecular chemistry. While bottom-up coarse-grained simulation approaches typically require making a priori decisions about which mechanical details of the interacting monomers will be represented, then tuning those parameters until known topological effects emerge (e.g., emergence of protofilaments with a cross-beta structure, known as 1,2 2-ribbons in the parlance of NHMs), the top-down NHM approach casts the model in a purely topological description ab initio, whereby the few body interactions driving the self-assembly of the higher order structure can be read directly from the parametrized NHM. Just as bottom-up coarse-grained models can offer tremendous value in offering a potential minimal set of monomeric mechanical degrees of freedom necessary for producing known supramolecular assemblies, the NHM methodology offers a complementary value in proposing minimal sets of few body intermolecular interactions that are capable of producing higher order supramolecular structure as an emergent property.

Potential future research directions in this vein of research include applying this approach to optimizing network Hamiltonian models to explore potential mechanisms for other self-assembly processes: from other supramolecular assemblies of proteins, to microtubule formation, to perhaps even self-assembly at the cellular level. Potential broader impacts for this work are that our methodology may be a useful tool for the study of protein aggregation diseases as well as the rational design of engineered self-assembling nanostructures and polymers. It is worth noting that our genetic algorithm also offers utility for users developing novel network Hamiltonian models, as evolution that fails to converge on individual models capable of producing a significant amount of the desired higher order periodic structure can be taken as an indication that the chosen sufficient statistics are perhaps not, in-fact, sufficient for capturing this behavior. The inverse approach also has utility, in that users can evaluate whether all terms in an existing model are actually necessary by removing terms in question, allowing the parameters for the remaining terms to evolve, and determining whether more parsimonious models are possible. By offering a free open-source software implementation of our methodology for automated parametrization of network Hamiltonian models, the authors aim to lower the barrier to increased adoption of this simulation methodology for studying self-assembly phenomena. The code used to generate the present work is available for download via the lead author’s GitHub page.^76,77^

## Supporting Information Available

R code implementation of the genetic algorithm for network Hamiltonian model parameterization (ZIP)
